# The Transcription Factor-microRNA Regulatory Network during hESC-chondrogenesis

**DOI:** 10.1038/s41598-020-61734-4

**Published:** 2020-03-16

**Authors:** Rosie Griffiths, Steven Woods, Aixin Cheng, Ping Wang, Sam Griffiths-Jones, Matthew Ronshaugen, Susan J. Kimber

**Affiliations:** 1 Divisions of Cell Matrix Biology and Regenerative Medicine, Faculty of Biology Medicine and Health, Michael Smith Building, Oxford Road, University of Manchester, Manchester Academic Health Science Centre, Manchester, M13 9PT UK; 2 Evolution and Genomic Science, Faculty of Biology Medicine and Health, Michael Smith Building, Oxford Road, University of Manchester, Manchester Academic Health Science Centre, Manchester, M13 9PT UK; 3 Developmental Biology and Medicine, Faculty of Biology Medicine and Health, Michael Smith Building, Oxford Road, University of Manchester, Manchester Academic Health Science Centre, Manchester, M13 9PT UK; 4 Present Address: Centre for Discovery Brain Sciences, University of Edinburgh, Hugh Robson Building, George Square, Edinburgh EH8 9XD United Kingdom; 5 Present Address: Salford Royal NHS Foundation Trust, Department of Trauma and Orthopaedic, Stott Lane, Salford M6 8HD United Kingdom

**Keywords:** Pluripotent stem cells, Stem-cell research

## Abstract

Human embryonic stem cells (ESCs) offer a promising therapeutic approach for osteoarthritis (OA). The unlimited source of cells capable of differentiating to chondrocytes has potential for repairing damaged cartilage or to generate disease models via gene editing. However their use is limited by the efficiency of chondrogenic differentiation. An improved understanding of the transcriptional and post-transcriptional regulation of chondrogenesis will enable us to improve hESC chondrogenic differentiation protocols. Small RNA-seq and whole transcriptome sequencing was performed on distinct stages of hESC-directed chondrogenesis. This revealed significant changes in the expression of several microRNAs including upregulation of known cartilage associated microRNAs and those transcribed from the Hox complexes, and the downregulation of pluripotency associated microRNAs. Integration of miRomes and transcriptomes generated during hESC-directed chondrogenesis identified key functionally related clusters of co-expressed microRNAs and protein coding genes, associated with pluripotency, primitive streak, limb development and extracellular matrix. Analysis identified regulators of hESC-directed chondrogenesis such as miR-29c-3p with 10 of its established targets identified as co-regulated ‘ECM organisation’ genes and miR-22-3p which is highly co-expressed with ECM genes and may regulate these genes indirectly by targeting the chondrogenic regulators SP1 and HDAC4. We identified several upregulated transcription factors including HOXA9/A10/D13 involved in limb patterning and RELA, JUN and NFAT5, which have targets enriched with ECM associated genes. We have developed an unbiased approach for integrating transcriptome and miRome using protein-protein interactions, transcription factor regulation and miRNA target interactions and identified key regulatory networks prominent in hESC chondrogenesis.

## Introduction

Human embryonic stem cells (hESCs) and induced pluripotent stem cells (iPSCs) hold great promise for regenerative medicine, due to their ability to undergo unlimited self-renewal and to respond to external stimuli directing their differentiation into cell types of all three germ layers (pluripotency). As a result, hESCs and hiPSCs are increasingly being used to model and understand normal and disrupted developmental processes in humans, particularly monogenic developmental diseases. However, the usefulness of such pluripotent stem cells is contingent on understanding the molecular basis of their *in vitro* differentiation in relation to normal development.

In the skeletal field new therapeutic approaches to treat joint diseases such as osteoarthritis (OA) and sports injuries are urgently needed since neither joint replacement nor autologous chondrocyte implantation is suitable for all individuals.

A number of groups have now developed differentiation protocols capable of directing pluripotent stem cells to desired target cell types, including chondrocytes and chondroprogenitors^1–10^. Many of these protocols rely on generation of embryoid bodies, and MSC-like cells, or use serum. We have developed a three stage serum-free protocol to generate chondroprogenitors from hESCs in a 2D culture plate, mimicking the early stages of normal chondrocyte development^11^. After 14 days *in vitro* the chondroprogenitors are able to contribute to high quality cartilage repair of an osteochondral defect in the trochlear groove of the hind limb femoral head of immunocompromised rats, with human cells still present in the defect repair area after several months^12,13^.

In order to use such cells for human joint repair it will be critical to fully understand the molecular networks and pathways driving their differentiation down the chondrogenic lineage into cartilage. This understanding may lead to modifications of the protocol resulting in more rapid, efficient, and complete differentiation. Cartilage is 20–35% ECM proteoglycan and protein and 70–80% water^14^ with chondrocytes, which synthesise the matrix, making up about 3% of the tissue and being the only cells present. MicroRNAs are increasingly identified as key players in regulating cell phenotypes through inhibiting translation or inducing mRNA degradation in many differentiation processes, including for skeletal development and ECM^15–18^. Their role in cartilage development is critical: the loss of microRNAs or inhibition of their processing causes cartilage development defects in mice and chondrocyte death^19,20^. Furthermore, previous large-scale small RNA profiling experiments have identified several microRNAs and microRNA-target interactions as regulators of the pathogenesis of osteoarthritis^17,21–24^, human cartilage development^25^ and chondrogenesis^26,27^. MicroRNAs show great promise as molecules for both therapeutic treatments and modulating differentiation processes^17^. Consequently, understanding miRome transcriptome interaction is critical to improving *in vitro* pluripotent stem cell (PSC) chondrogenesis for both cell therapy and disease modeling applications.

We have undertaken a high-throughput RNA-seq analysis of the transcriptome and miRome of hESCs progressing through our directed differentiation protocol to chondroprogenitors. Using a systems biology approach to understand the transcriptome-miRome relationship we revealed networks of known chondrogenic gene activity. Furthermore we identified gene and microRNA regulatory modules with potentially undiscovered roles in human cartilage development. These chondrogenesis-associated factors constitute targets that may prove useful for further optimization of differentiation protocols to produce chondrocytes for cartilage repair and osteoarthritis treatment or disease modeling from iPSCs.

## Results

### Global transcript and microRNA changes during hESC chondrogenesis

**Figure 1:**
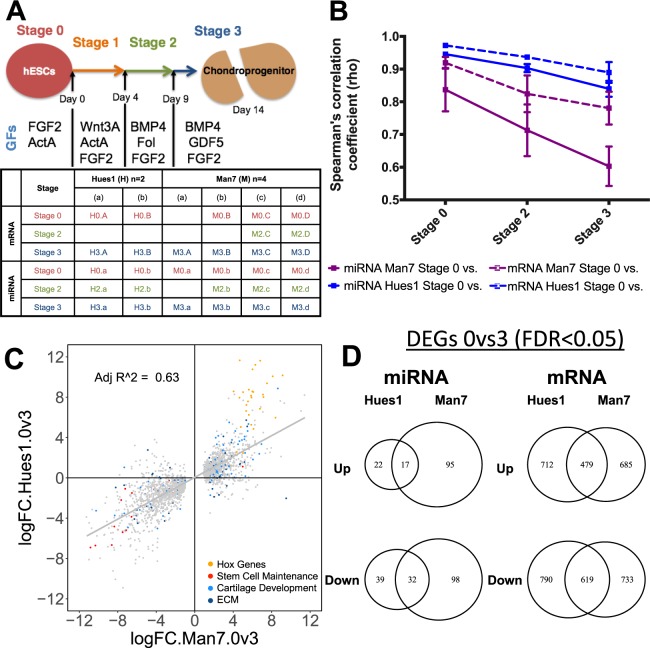
Global miRome and transcriptomic changes during hESC chondrogenesis. (**A**) Time course, top, and experimental plan, bottom, showing when samples were taken and how they were labelled. Top, Cells were differentiated using a series of growth factors over 14 days as in Materials and Methods, ActA, activin A; Fol, Follistatin. (**B**) Average Spearman’s correlation coefficients (y-axis) comparisons between stage 0 with all stages of the DDP (x-axis). Coloured lines indicate cell line, dotted line or solid line represent miRome or transcriptome, respectively. Error bars represent the standard deviations of the coefficients for all replicates in the comparison. (**C**) X-Y scatter graph of the log2 fold change between stage 0 and 3 of genes differentially expressed in Man7 (x-axis) and Hues1 (y-axis) (FDR < 0.05). (**D**) Venn diagram showing overlap of differentially expressed genes and miRNAs in both hESC lines.

To investigate the key pathways driving hESC chondrogenesis, we differentiated two hESC lines (Man7, n = 4; Hues1, n = 2) into chondroprogenitors using the directed differentiation protocol (DDP) as previously described^11,12^. Cells progress through several distinct stages of differentiation, initially through a primitive streak/mesendoderm population (stage 1), then towards a (lateral) mesoderm state (stage 2), and finally acquiring a chondrogenic progenitor phenotype (stage 3). The increased expression of the cartilage markers *SOX9* and *COL2A1*, along with loss of pluripotency associated genes *OCT4* and *NANOG*, during the differentiation process, was validated by RT Q-PCR (Fig. S1). Small RNA and whole transcriptome libraries were generated and RNA-seq performed, for samples collected at the end of stages 0 (hESC), 2 (mesoderm) and 3 (chondroprogenitor) of the protocol (Fig. 1A)^11^.

Mapping the transcriptome reads (Table S1) to the human genome (hg19) shows the expression of 22,073 transcripts (Table S2), 17,835 of which are annotated as protein-coding genes according to Ensembl. For the small-RNA seq libraries 1,052 mature microRNAs were detected (Table S2). A Spearman’s correlation matrix was generated to investigate the degree of variability between samples during the protocol (Fig. S2). As expected, both the miRomes and transcriptomes of the hESCs became increasingly divergent from stage 0 during the differentiation process as indicated by the decreasing Spearman’s correlation coefficients (Figs. S2, 1B). Although, the transcriptome and miRome of Man7 changed more during differentiation than that of Hues1, there was a good degree of correlation in transcript expression changes between stage 0 (pluripotent) and stage 3 (chondroprogenitor) for the two lines (Fig. 1C). Furthermore, there were 479 significantly upregulated transcripts and 619 significantly down regulated transcripts in common between the two lines (Fig. 1D).

Principal component analysis (PCA) of both the whole transcriptome and miRome showed a clear progression from stage 0 to stage 3 with stage 2 being intermediate (Fig. S3). Stage 2 samples show greater variation than other stages, likely due to more inter-cell variability at this dynamic stage of differentiation. The small RNA-seq library preparation appears to be more sensitive to batch variation than the whole transcriptome data (Fig. S3, shown in miRome PCA, with the two batches separating out above and below the grey arrow).

### Transcriptome and miRome regulation during hESC chondrogenesis

**Figure 2:**
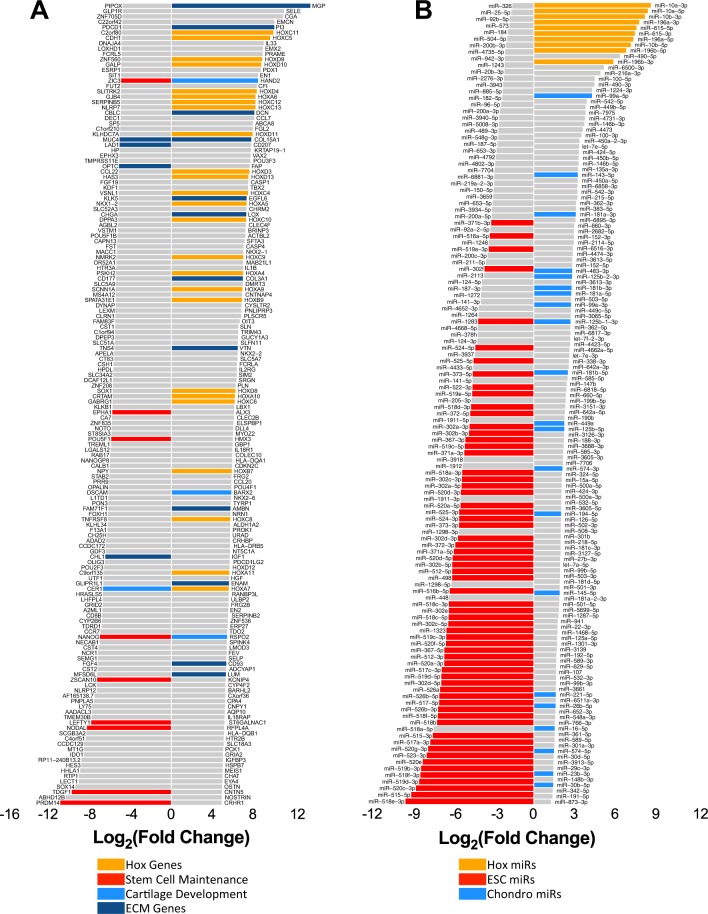
Differential expression analysis between hESCs and chondroprogenitors. Top 150 up-regulated and 150 down-regulated transcripts (**A**) and all significantly differentially expressed microRNAs (**B**) between hESCs and chondroprogenitors (FDR < 0.05). Some bars are highlighted by function identified from the literature.

Differential expression analysis between hESCs and chondrogenic samples identified 3274 significantly regulated transcripts (FDR < 0.05) (Table S4), 2635 of which are annotated as protein-coding genes. The top 150 up and down regulated genes between hESCs and chondroprogenitors are shown in Fig. 2, whilst the up and down regulated genes at the intermediate stage 2 are shown in Fig. S4. Some of the most up-regulated genes during hESC-chondrogenesis were Hox genes (particularly the HOX A, C and D clusters), ECM associated genes (LOX, DCN, MGP and COL15A1) and transcription factors involved in mesodermal and limb development (BARX2, HAND2 and TBX2^28–30^; (Fig. 2A and Table S4). The most down-regulated genes were pluripotency-associated genes (NANOG, POU5F1, NODAL, LEFTY1, ZSCAN10) (Fig. 2A and Table S4). We also identified 727 and 48 differentially expression genes between the intermediate stage and hESC or chondrogenic samples respectively (Table S4 and Fig. S4).

Differential expression analysis identified 208 microRNAs that exhibited significant changes in expression between hESCs and chondroprogenitors (Fig. 2B, Table S3). There was a down-regulation of many pluripotency-associated microRNAs (Fig. 2B, highlighted in red) mainly from a large cluster on chromosome 19 (CM19C), but also the miR-302 family^31,32^. Conversely microRNAs transcribed from the Hox gene cluster, which regulate HOX gene function, were among the most up-regulated (Fig. 2B; orange), together with several microRNAs that have been reported to have a role in chondrogenesis or cartilage maintenance such as miR-99a-5p, miR-143-5p and miR-181a-3p (Fig. 2B; blue). There was a good correlation between RNAseq and Q-PCR validation data (Fig. S5).

Gene Ontology analysis of transcripts differentially expressed between hESCs and chondroprogenitors identified expected enrichment of GO terms ‘skeletal system development’ as well as ‘cartilage development’ and ‘extracellular matrix organisation’ to be enriched (Table S5).

**Figure 3:**
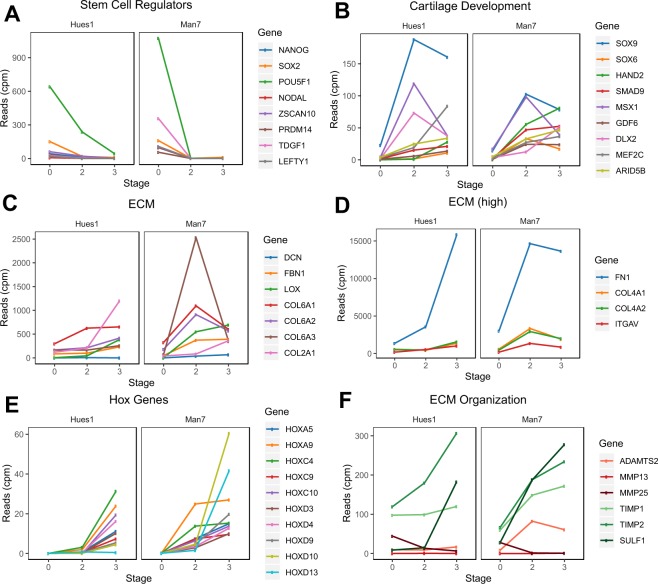
Expression of key regulated genes during hESC-chondrogenesis. Expression during directed chondrogenesis of hESCs, of known (**A**) stem cell regulators, (**B**) cartilage development regulators, (**C**) ECM genes, (**D**) highly expressed ECM genes, (**E**) Hox genes, (**F**) positive ECM modifiers (green shades) and negative ECM regulators (red shades).

Extraction of genes within these terms shows that a number of those associated with cartilage development peaked at the intermediate stage of differentiation (SOX9, MSX1 and DLX2) after which they were down-regulated (Fig. 3B). At the later stage of differentiation, several of the ECM genes became up-regulated (Fig. 3C–F): for example, high expression of positive regulators of ECM maintenance/organization (TIMPs and SULF1) (green, Fig. 3F) was noted, while ECM proteases had low expression (red, Fig. 3F).

**Figure 4:**
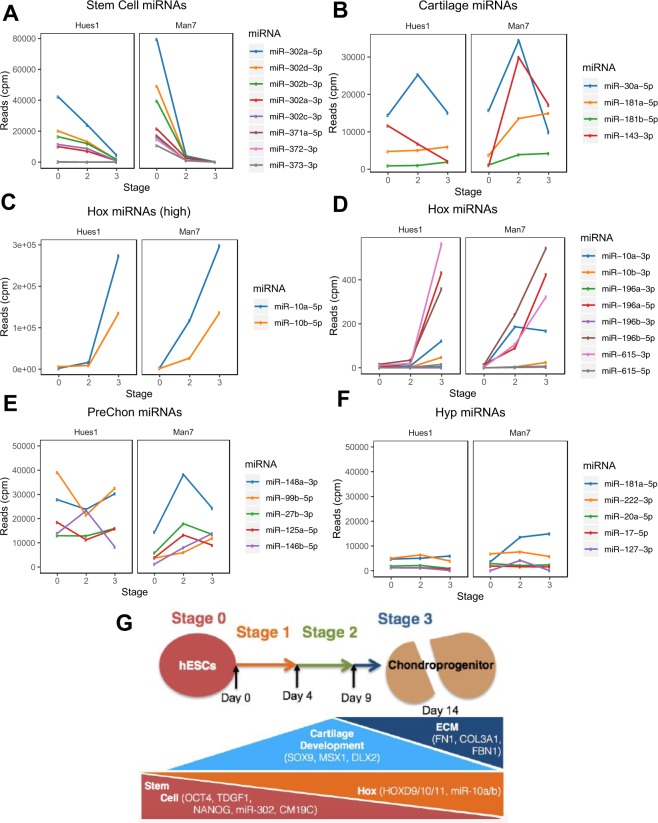
Expression of key regulated miRNAs during hESC-chondrogenesis. Expression during directed chondrogenesis of hESCs, of known (**A**) stem cell-associated microRNAs, (**B**) microRNAs that regulate cartilage development, (**C**,**D**) Hox complex microRNAs, (**E**) microRNAs reported to be upregulated in prechondrocytes, (**F**) microRNAs reported to be upregulated in hypertrophic chondrocytes. (**G**) Summary of transcript and microRNA expression changes during hESC chondrogenesis.

The miRome expression patterns show similar trends to the whole transcriptome data (Fig. 4). The majority of stem cell microRNAs were down-regulated during early differentiation, although miR-302b-5p, was only lost during late differentiation in Hues1 (Fig. 4A). Some known cartilage microRNAs and Hox microRNAs were subsequently up-regulated (Fig. 4B–D) as expected. Previous work defined a number of precursor chondrocyte microRNAs and those expressed in hypertrophic chondrocytes^25^. Figure 4E,F show that there is much higher expression of prechondrocyte microRNAs (e.g. miR 99b-5p; 27b-3p) than hypertrophic chondrocyte-associated microRNAs (miR 20a-5p,17-5p and 127-3p) during the hESC-chondrogenesis protocol. A summary of key changes in transcript and microRNA expression during hESC chondrogenesis is shown in Fig. 4G.

### Discovery of novel chondrogenic genes and microRNAs

**Figure 5:**
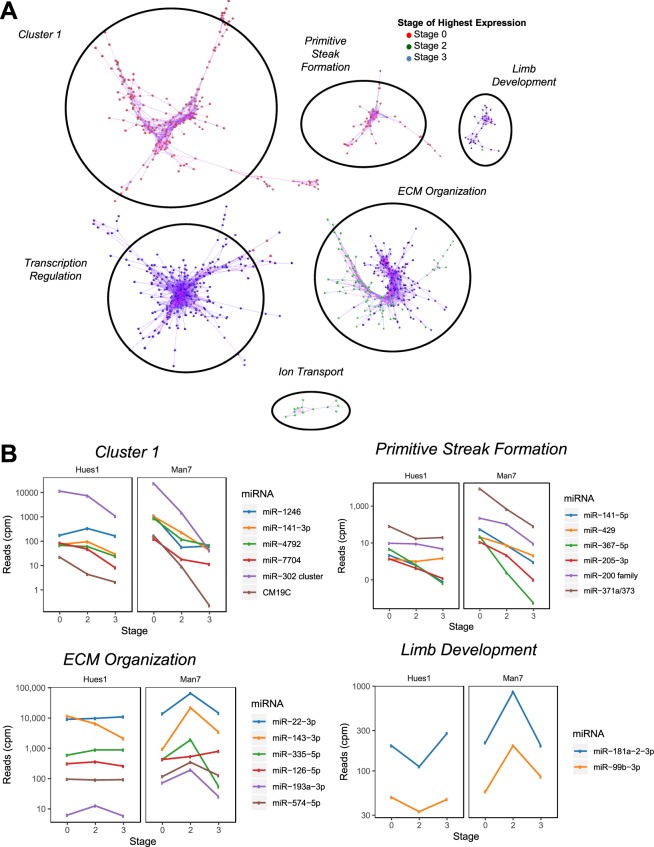
Co-expression Network analysis of microRNAs and mRNAs during hESC-chondrogenesis. (**A**) Pearson’s correlation analysis of all genes and miRNAs expressed during hESC-directed chondrogenesis. Top clusters of miRNAs and mRNAs using Biolayout with a Pearson’s correlation of >0.98. Each node represents a gene or miRNA with connecting lines representing their correlation with other genes/miRNAs. Nodes are highlighted based on their stage of highest expression with values averaged over cell lines and replicates. (**B**) Expression during directed-chondrogenesis of Man7 and Hues1 of the top expressed microRNAs within Cluster 1 (top, left), Primitive Streak cluster (top, right), ECM cluster (bottom, left) and Limb cluster (bottom, right) during differentiation.

To uncover novel chondrogenic microRNAs and genes from the RNA-seq data we employed a co-expression strategy on the assumption that genes expressed with a similar pattern to previously identified chondrocyte genes may be either regulated by these chondrocyte genes, regulators of these genes, or regulated by a similar mechanism. Pearson’s correlation of gene and microRNA read counts using BioLayout^33^, independent of DDP stage, identified six clusters of co-expressed genes and microRNAs (Figs. 5A, S7 and Table S6). GO term enrichment analysis was performed on each of the six clusters (Table S7).

Although there was no significant association (FDR < 0.05) of any GO term with cluster 1, it contained genes highly expressed in hESCs and down-regulated during the DDP some of which are associated with stem cell maintenance (NANOG, POU5F1 and NODAL), or known to regulate the stem cell state (LEFTY1, LEFTY2 and ZFP42)^34,35^. Cluster 2 and 3 were most enriched for ‘primitive streak formation’ (fold enrichment >100, FDR = 0.000144) and ‘regulation of transcription by RNA polymerase II’ (fold enrichment 2.1, FDR = 9.8 × 10-7) respectively (Table S7). Clusters 4 and 5 were significantly enriched with a number of cartilage related GO terms; for example ‘extracellular matrix organization’ (fold enrichment 9.1, FDR = 4.5 × 10^−24^) and ‘limb development’ (fold enrichment 19.4, FDR = 9.5 × 10^−5^) (Table S7). The average read counts for the ‘extracellular matrix organization’ and ‘limb development’ clusters were higher in Man7 than Hues1 at all stages during differentiation. Expression levels of genes within the ‘ECM organization’ cluster and ‘limb development’ clusters peaked earlier in Man7 than Hues1 (Fig. S7), possibly indicating Man7 has a greater capacity to produce cartilage or is more responsive to the chondrogenic differentiation protocol. There was an association of ‘ion transport’ with cluster 6, although this did not reach FDR significance of <0.05.

Expression changes for selected transcripts and miRNAs and are shown in Fig. 5B. MicroRNAs in each cluster appeared to have generally similar functions to the co-expressed protein coding genes. For example, the well-documented pluripotent stem cell microRNA, miR-302a^32,36^ was in cluster 1. MicroRNAs miR-200 and miR-141, known regulators of the epithelial-to-mesenchymal (EMT) transition^37^ are in the ‘Primitive Steak Formation’ cluster reflecting the dependence of primitive streak formation on EMT. It was notable that the two limb development cluster microRNAs miR-181a-2-3p and miR-99b-3p showed a similar pattern in the two lines. Of the microRNAs in the ‘ECM Organization’ cluster, 11 out of 24 (miR-145-5p, miR-143-3p/5p, miR193a-3p, 199a/b-3p, miR-29a-5p, miR-335-5p, miR-574-3p/5p, miR675-3p/5p) have been reported to regulate chondrogenesis (Table S6)^27,38–45^. However, the most highly expressed microRNA in this cluster, miR-22-3p, may be a novel regulator of chondrogenesis (Fig. 5B). There were 26 microRNAs within the ‘transcription regulation’ cluster and no miRNAs within the ‘ion transport’ cluster (Table S6).

### Key regulators of hESC directed chondrogenesis

**Figure 6:**
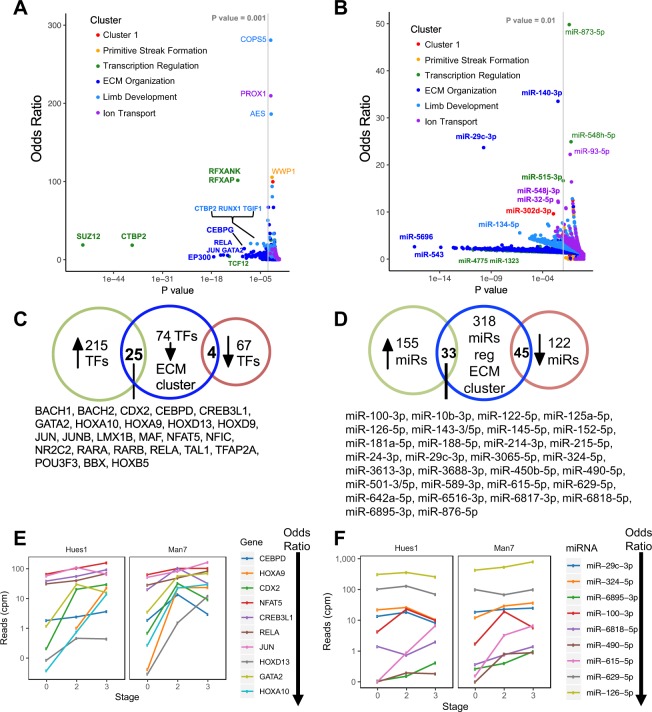
Transcription factors and microRNAs regulating co-expressed clusters of genes during chondrogenesis. X-Y scatter plot showing the odds ratio (y-axis) and P-value (x-axis) for the enrichment of targets of each TF (**A**) or microRNA (**B**) within a co-expressed cluster calculated using a Fisher’s Exact Test. (**C**) Venn diagram showing overlap of TFs (**C**) or microRNA (**D**) with an enrichment of targets within the ECM cluster (P-value < 0.001 and Odds ratio >1) (center) and if they are significantly up-regulated (left) or down-regulated (right) during hESC-chondrogenesis. Expression of significantly upregulated TFs (**E**) or microRNA (**F**) with the highest enrichment of targets within the “ECM Organisation’ cluster during differentiation.

We next focused on transcription factors (TFs), as high-level regulators of differentiation. We identified the TFs that have enriched targets within one or more of the clusters of co-expressed genes, using a database of known TF-target interactions^46,47^ and evaluated significance using a Fishers Exact Test. For each TF we calculated an odds ratio by comparing the ratio of TF target genes to non-target genes within each cluster versus the ratio of TF target to non-target genes not in the cluster: an odds ratio >1 indicates an enrichment of TF targets within the cluster of genes. This enrichment analysis showed that 3 of the 6 clusters (Limb development, ECM organisation, Transcription regulation) showed significant enrichment for one or more transcription factors (Fig. 6A, Table S8). We identified 77 TFs with target enrichment within the ‘ECM Organization’ cluster of genes (odds ratio > 1, P value < 0.001), 25 of which are significantly up-regulated during hESC-chondrogenesis (Fig. 6C). Several of these TFs show a progressive upregulation during hESC-chondrogenesis (Fig. 6E), including JUN and RELA which target a large number of genes within the ‘ECM Organization’ cluster (39 and 36 respectively, Table S8) including several ECM modifying genes (TIMP1, LOX, TGM2 and ADAM9) and ECM genes (COL1A1, COL1A2, COL4A2, DCN, FN1 and HSPG2). Taken together, the data suggest that there is a strong regulatory drive during human chondrogenic differentiation to synthesize ECM components prevalent in cartilage and we have identified several TFs potentially regulating this process, some of which have not previously been associated with chondrocyte regulation.

**Figure 7:**
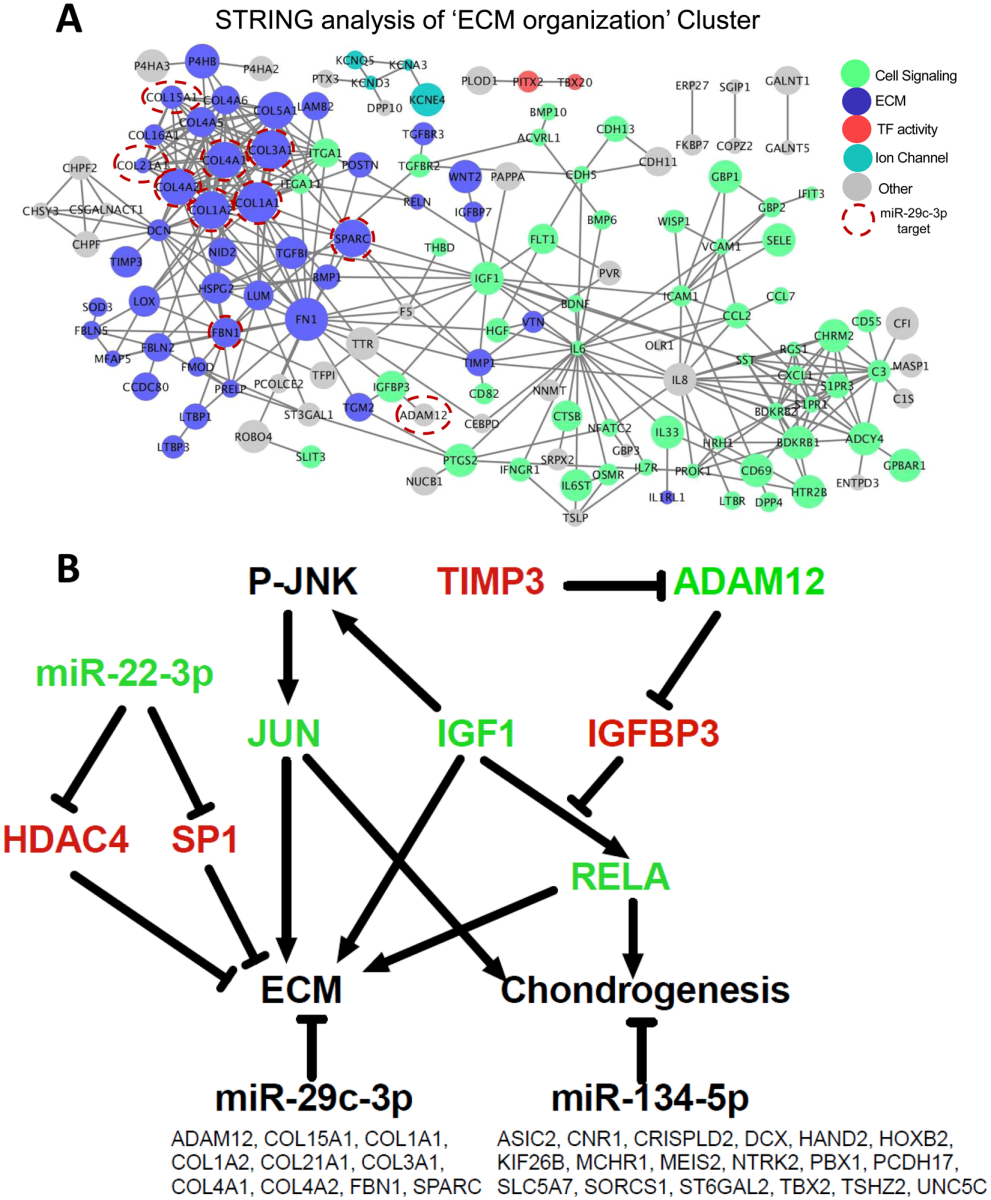
Protein interactions for ECM and summary of regulators. (**A**) Protein-protein interaction network for the ‘ECM Organisation’ cluster of co-expressed genes generated using STRING. Genes have been highlighted using GO terms. (**B**) Summary of key chondrogenic regulators identified and their relationship to known regulators. Pathway of key regulators of hESC-chondrogenesis identified in our analysis using a variety of system biology approaches along with known interactions from the literature. IGF1 is highly co-expressed with several ECM organization associated genes during hESC-chondrogenesis and is an upstream regulator of RELA and JUN. Both these TFs have targets enriched within the cluster of ECM organization genes. Along with IGF-1, miR-22-3p is also highly co-expressed with ECM organization genes during the hESC-differentiation and may be promoting hESC-chondrogenesis by targeting HDAC4 and SP1. MiR-29c-3p and miR-134-5p were also identified as chondrogenic regulators as they had a significant enrichment of targets within the ECM organization cluster and limb development cluster, respectively (targets shown below each microRNA). Molecules in green font are positive regulators of chondrogenesis while those in red are negative regulators of chondrogenesis.

We next applied the same approach to find microRNAs which have targets enriched within each cluster. A list of high-confidence miR-target interactions was generated by combining high scoring predicted interactions from the TargetScan database (total context score < −0.3) with miRTarBase data filtered for only luciferase reporter assay validated interactions. Similarly to the TF analysis, all of the clusters were enriched with targets of one of more microRNAs (Fig. 6B, Table S9). The ECM cluster also contained the most enriched targets of microRNAs (Fig. 6B,D), with miR-29c-3p and miR-140-3p targets most enriched (odds ratios of 33.5 and 23.7 respectively; FDR < 0.05). Although miR-29c-3p was identified by analysis independent of our microRNA expression data, miR-29c-3p was in fact increased in expression (Fig. 6F): similar to the genes which it is predicted to target within the ECM organization cluster. Ten of the 39 established miR-29c-3p targets are part of the ‘ECM organisation’ cluster (Table S10, Fig. 7A). Targets of miR-134-5p are 5.5-fold enriched in the limb development cluster, many of which are known limb developmental regulators (HAND2, HOXB2, MEIS2, PBX1 and TBX2).

### Protein-protein interaction analysis of ECM organization cluster

Mechanisms maintaining the network of positive and negative ECM regulators may be essential for healthy cartilage homeostasis. A protein-protein interaction network for the ‘ECM Organization’ cluster was generated (Fig. 7A) using the STRING repository of protein-protein interactions^48^. The resulting network had a significant enrichment of protein-protein interactions (p < 1 × 10^−16^) and contained several cell-signaling molecules, with IL6 identified as a key gene connecting several sub-networks together. A sub-network of collagens and genes involved in metabolism of the most prevalent cartilage GAG, chondroitin sulfate, are also present. A network of genes linked to IGF1, a positive regulator of cartilage ECM^49–52^, was identified within this network, including IGFBP3. Transcripts for the cell adhesion molecules CDH5,11 and 13, identified by STRING analysis, are also known to be expressed in the chick limb bud with CDH11 present from an early stage^53^. It is likely that the potassium voltage gated ion channels identified (KCNQ5, KCNA3, KCNE4, KCND3) may similarly be involved in ion balance and mechano-regulation. Nine of the 10 miR-29c-3p targets within the ‘ECM organization’ cluster are within a sub-cluster of ECM proteins (Fig. 7A).

In Fig. 7B we summarise the major new players associated with hESC-chondrogenesis which we have extracted from our analysis.

## Discussion

Human limb and cartilage development are extremely difficult to study for obvious ethical reasons, so our understanding of limb bud development, endochondral skeleton formation and generation of the permanent cartilage has relied largely on study of chick and mouse embryos. To understand human cartilage development researchers have turned to human mesenchymal stromal cells (MSCs). MSCs have been informative but they do not recapitulate normal development of the permanent cartilage and the chondrocytes produced tend to generate a fibrocartilage or become hypertrophic^54,55^. Several groups including ourselves^11–13,56^ have therefore exploited the plasticity of human pluripotent stem cells to obtain more authentic cartilage development. In this report we demonstrate that this approach can lead to cells with characteristics of embryonic limb bud and later chondrogenic precursors even in a 2D culture format.

### Characterisation of hESC- chondroprogenitors

Using an unbiased approach we characterised chondroprogenitors derived from two different hESC lines. As expected the differentiating cells showed a general increase in Hox genes, in particular in the Hox D (HOXD4, D10, D9, D11, and D13) and C clusters (HOXC4, C9, C11 and 12), as well as HOX A5, A6 and A9. HOXD cluster genes have been implicated in mesenchymal condensation. HOXA and D cluster appear to pattern different elements of the forelimbs while the HOXC cluster patterns the hindlimbs^57^. For instance, HOXD9, D12 and D13 play roles in patterning of chick and murine limb bud^57–59^. HOXD13 and D11 are both involved in long bone growth and bone length^58^. HOXA9 together with HOXD13 has also been shown to be important for orchestrating limb regeneration^60^ based on experiments in axolotls. Expression of these HOX genes indicates that some of the regulatory machinery implicated in limb development is expressed in our protocol. During differentiation to intermediate cells, expression of stem cell regulators is lost while regulators of limb development are up-regulated (DLX2, DLK1, MSX1, SOX9, SOX6). The dramatic upregulation of SOX9, the master regulatory transcription factor of chondrogenesis^61,62^, was followed by a slight decline, which reflects the need for concerted initiation of transcription for ECM molecules but then a lower transcription required to maintain production. The chondroprogenitors express high levels of the ECM genes (COL3A1, COL4A1, COL4A2, COL6A1, COL6A2, COL6A3, FN1, FBN1) with FN1 being the highest expressed transcript. Notably Fibronectin^63^, Collagen IV and Collagen VI are found in the pericellular matrix of cartilage^63,64^ suggesting that these chondroprogenitors are assembling a pericellular matrix prior to later chondron formation.

These chondroprogenitors also have low expression of ECM metalloproteases (MMP13 and MMP25) while their inhibitors are highly expressed (TIMP1 and TIMP2), indicating anabolic matrix production. The high expression of TIMPs and SULF1, a heparan sulfate 6-O-endo-sulfatase in the chondroprogenitors reflects the central requirement for net ECM synthesis and GAG modification during chondrogenesis. Furthermore, RUNX2 is low and the hypertrophic marker COL10A1 is absent, indicating absence of growth plate-like chondrocytes. Several other ECM genes, less frequently reported in limb and cartilage development, are also up-regulated (DCN, MGP and LUM)^65–68^.

Some of the main constituents found in the ECM of articular cartilage are expressed in the chondroprogenitors (COL2A1, ACAN, and COMP), but not at high levels. This may be due to the limitation of using a 2D culture system and we hypothesize that if chondroprogenitors from this protocol were to be cultured on in a 3D format they would further up-regulate markers of chondrogenesis as indicated by others (13,14 and our unpublished data).

### Comparison with developing cartilage

In our system we see an early up-regulation of cartilage development regulators (HAND2, DLX1/2, MSX1 and PTHLH). MSX1 and DLX2 are co-expressed in the developing mouse limb bud mesenchyme^69^. Although it was not possible to do a systematic analysis, comparison of our data with the limited available transcriptomic analysis of developing human cartilage shows large overlap. Several genes expressed in prechondrocytes micro-dissected from 5–6 week developing limb were also up-regulated in our system including the transcription factors FOXC1, FOXP2, SOX5/6/9, GLI3, HOXD10/D11^70^. Furthermore a number of receptors up-regulated in 17 week resting fetal chondrocytes compared to prechondrocytes were also up-regulated in our chondroprogenitors (CD59, CD82, CD55, CD109 and ICAM1)^70^. In our study the expression of these genes was up-regulated between the intermediate stage and the chondroprogenitors confirming the further progression of chondrocyte differentiation.

Most of the published data on microRNA regulation of human chondrogenesis is taken from MSC differentiation, which has the potential disadvantages in terms of developmental progression and type of cartilage suggested above. However in a few cases developing human cartilage has been obtained for analysis. One study used laser microcapture dissection to isolate three regions of developing human cartilage (precursor chondrocytes, differentiated chondrocytes and hypertrophic chondrocytes) and performed microRNA profiling on each. Of the microRNAs up-regulated in our hESC-chondrogenesis differentiation protocol, 27 were also differentially expressed between precursor chondrocytes and hypertrophic chondrocytes^25^. Indeed 25 of these also showed up regulation in precursor chondrocytes in the McAlinden study confirming the generation of prechondrocytes in our protocol.

### Identified chondrogenic microRNAs

The dynamic change in microRNA expression documented in this study, demonstrate how these molecules may prove to be more useful as biomarkers of developmental progression than mRNAs or proteins: they show more dynamic changes in expression compared to mRNAs. A number of chondrogenic microRNAs were up-regulated in the chondroprogenitors (miR-30a, miR-181a, miR-143, miR-99a and miR-483) along with several that may be novel regulators as they have yet to be reported to regulate chondrogenesis. For example, miR-490 which increases during differentiation, has been reported to target several members of the TGFβ signaling pathway, including TGFβR1^71^ and BMPR2^72^. miR-100 is also highly expressed in OA cartilage^24^ and down-regulated in chondrosarcoma compared with articular chondrocytes^73^. miR-100 can inhibit MSC-osteogenesis by targeting BMPR2^74^.

### Identifying novel chondrogenic regulators

Here we have utilised a co-expression method to identify potential novel functionality of transcripts and microRNAs dynamically regulated during chondrogenesis. We have identified six clusters of co-expressed genes and microRNAs each enriched with genes regulating a specific process in hESC-chondrogenesis. The second largest co-expressed cluster (after transcription regulation) contained genes regulating ECM organisation, in agreement with the key role of ECM in cartilage.

Several of the microRNAs within the clusters have similar functionality to the transcripts they are co-expressed with. For example cluster 1 containing pluripotency transcription factors (NANOG and POU5F1) includes the miR-302 family, known regulators of pluripotency maintenance^32^. MiRNAs within the ECM organization cluster provide another potential cohort of novel chondrogenic regulatory microRNAs. The highest expressed microRNA within the ECM organisation cluster is miR-22-3p (Fig. 6B). This was also identified in human osteoarthritic cartilage^21^. Notably miR-22-3p has been validated to target several chondrogenic regulators including HDAC4^75^, SP1^76^ and SIRT1^76,77^. HDAC4 and SP1 are also both targets of the well-known cartilage microRNA miR-140^78,79^. This data suggests miR-22-3p may be acting in a similar way to miR-140 in chondrogenesis, to promote ECM synthesis by inhibiting hypertrophy via targeting of HDAC4^80^ and by preventing ECM degradation by its targeting of SP1 which induces ADAMTS-4 expression^81^.

These clusters of genes and microRNAs were further investigated using a systems biology approach. By using known TF-target interactions^46^ we identified 25 TFs that are upregulated during hESC-chondrogenesis and enriched with targets involved in ECM organisation. Some of the TFs identified have previously been reported to regulate chondrogenesis. For example, JUN has been reported to both positively^82^ and negatively regulate this process^52,83^. RELA has been shown to promote growth plate chondrogenesis^84^; interestingly, RELA is downstream of IGF-1 signaling^85^ a central feature of the ECM organization network. We also identified several TFs (SUZ12, RUNX1, CTBP2 and TGIF1) targeting limb development regulators.

We used the same methodology to identify which microRNAs may be regulating expression of the gene clusters by microRNA-target interactions. This identified several microRNAs which had an enrichment of targets within the ECM organisation cluster, including the most well reported cartilage microRNA miR-140-3p, members of the miR-29 family (miR-29c-3p and miR-29a-5p) which have already been reported to regulate ECM^23,86^.

Furthermore, 9 of the 10 miR-29c-3p targets within the ‘ECM organization’ cluster’ in our data are within a sub-cluster of genes encoding ECM proteins, indicating that miR-29c-3p predominantly functions to directly control ECM protein genes during PSC chondrogenesis, with the exception of ADAM12. This is confirmed in an independent study, where inhibition of miR-29a in human dermal fibroblasts led to an upregulation of ECM genes enriched with the same ‘extracellular matrix organization’ GO term^87^ (Table S10). Interestingly 9 of the 11 genes contributing to this enrichment are predicted miR-29 targets^88^, suggesting miR-29 directly regulates ECM cluster genes in human fibroblasts.

In contrast to the commonly observed inverse correlation between miRNAs and their targets (for example miR-302d-3p in our data), miR-29-3p increases during differentiation but its targets tend not to decrease. Therefore miR-29-3p either acts to stabilise transcript expression, as previously observed for miR-322 within cartilage^89^, or miR-29 forms part of a complex regulatory network with matrix genes. MiR-134-5p had an enrichment of targets within the limb development cluster including HAND2, HOXB2, MEIS2, PBX1 and TBX2 all expressed by the chondroprogenitors.

By integrating our data with known protein-protein interactions^48^, key genes of the network were identified, including the signalling molecules IL6, IL8 and IGF-1. IL6 is strongly expressed in OA chondrocytes^90^ and circulating IL6 is a biomarker for OA^91^. OA involves ECM catabolism through the activity of matrix metalloproteinases (MMPs). TIMP1 and TIMP3, inhibitors of MMPs, are positively correlated with IL6 in our study.

ADAM12 expressed by human chondrocytes^92^ cleaves insulin-like growth factor binding protein-3 (upregulated in hESC-chondrogenesis) and -5 (IGFBP-3/5). Degradation of these binding proteins leads to a higher bioavailability of IGF-1 and increased chondrocyte proliferation^93–95^.

Our analysis of hESC chondrogenesis has identified multiple levels of regulation. Transcription factors such as JUN and RELA are likely to control ECM gene expression. Many of the ECM encoding genes are likely to be modulated by microRNAs, in particular miR-29. MiR-22-3p is highly co-expressed with ECM genes and its known targets suggest it is likely to be a novel chondrogenic regulator.

## Conclusions

Our system provides a validated model of early human cartilage development to study gene and microRNA interactions *in vitro* and to understand diseases involving early aberrations in cartilage formation. We have generated an in-depth analysis of the changes in mRNA transcripts and microRNAs during hESC-chondrogenesis. Chondroprogenitors express high levels of ECM genes as well as some of the transcription factors already indicated as controlling chondrogenesis in mouse and expressed in human cartilage development. Using a systems biology approach, we have identified the key transcriptional and post-transcriptional regulators of chondrogenesis, some of which have not previously been reported.

## Methods

### Cell culture and directed chondrogenic differentiation

HUES1 and MAN7 hESCs lines were cultured as previously described^11,12^. Briefly, hESCs were cultured on Mitomycin C inactivated mouse embryonic fibroblasts (iMEFs), plated at 6 × 10^4^ cm^−2^ on 0.1% gelatin (Sigma) coated dishes 24 h previously. The hESCs were cultured in knockout Dulbecco’s modified Eagle’s medium (DMEM) supplemented with 20% (v/v) knockout serum replacement, 2 mM L-glutamine, 1% (v/v) non-essential amino acids (NEAA), 0.1 mM β-mercaptoethanol, 1% (v/v) penicillin/streptomycin (all Invitrogen) and 10 ng/ml FGF2 (Autogen Bioclear, Wiltshire, UK). They were passaged using TrypLE^TM^ (Invitrogen). For feeder-free culture, cells were lifted from the iMEF layers with TrypLE^TM^, and plated onto fibronectin-coated (Millipore) tissue culture flasks with feeder-free medium: 50:50 F12:DMEM (Lonza) supplemented with 2 mM L-glutamine, 1% NEAA, 0.1 mM β-mercaptoethanol, 1% penicillin/streptomycin, 0.1% bovine serum albumin (BSA) (Sigma), 1% (vol/vol) N2 supplement 2% (vol/vol), B27 supplement (both Invitrogen), 10 ng/ml activin A, 4 ng/ml neurotrophin 4 (Preprotech, London, UK) and 20 ng/ml FGF2^96^. The hESCs were differentiated in a basal medium (DMEM:F12, 2mM L-glutamine, 1% (vol/vol) ITS, 1% (vol/vol) non-essential amino acids, 2% (vol/vol) B27, 90 μM β-mercaptoethanol) supplemented with appropriate sequential addition of growth factors as indicated below and as previously described^11,12^. Wnt3a (25 ng/ml R&D Systems), Activin-A (reducing from 50–10 ng/ml, Peprotech) and BMP4 (40 ng/ml; Peprotech) were applied to differentiate the hESCs towards primitive streak-mesendoderm (day1–3), followed by BMP4 (40 ng/ml), Follistatin (100 ng/ml) (all Peprotech) to drive differentiation to mesoderm (day 4–8), finally GDF5 (20 rising to 40 ng/ml), FGF2 (20 ng/ml) and NT4 (2 ng/ml) (all Peprotech) were used to promote chondrogenesis (day 9–14). A diagrammatic representation of the protocol is shown in Fig. 1A. Cells entered the differentiation protocol at passage 27 for Man7 and passage 32 for Hues1.

### RNA Isolation and sample preparation

RNA samples were isolated using miRvana microRNA isolation kit (Life Technologies). Total RNA was collected at day 0 (stage 0 samples), day 9 (stage 2 samples), and day 14 (stage 3 samples) of the protocol, represent pluripotent, mesodermal and chondrogenic stage of the differentiation pathway as indicated in Fig. 1A and reported previously^11,12^. In order to carry out global microRNA and mRNA expression analysis during chondrogenesis of hESCs, two or more small RNA libraries and whole transcriptome libraries were generated for stages 0, 2 and 3 of the defined chondrogenic protocol. A total of 1000 ng of RNA was used for library preparation using the Illumina TruSeq Stranded mRNA Sample preparation kit for transcriptome libraries and Illumina TruSeq Small RNA Library Preparation kit for small RNA libraries. All samples were run on the Illumina HiSeq. 2000 with paired-end reads, generating on average 50 million high-quality sequencing reads per sample (Table S1).

### RNA sequencing analysis

Raw small RNA sequencing data was uploaded to the Galaxy server and quality of reads was assessed using the FastQC tool (version 0.63). Adapter sequences were then trimmed from the small RNA reads using the Clip tool (version 1.0.1) discarding reads shorter than 15 nucleotides. The trimmed reads were then uploaded to miRanalyzer (version 0.3), where the small reads were mapped to miRBase 20. Whole transcriptome libraries were mapped to the human genome (hg19) using Bowtie2^97^. Mapped read counts were then summarised to GenecodeV16 using htseq-count^98^. First reads with low expression were filtered out, keeping only reads with at least 4 counts in two samples or more. Next a normalization factor was applied, small RNA reads were normalized using upper-quartile method and whole transcriptome was normalized using TMM method. Differential expression analysis was performed with the R package edgeR using a GLM-based likelihood ratio test^99^. Tags were considered differentially expressed if their corrected p-value was less than 0.05 (Benjamini-Hochberg correction). Gene ontology pathway analysis was performed using ClueGo (v2.3.2) with the Biological Processes ontology. Terms were considered significant if Bonferroni corrected p-value was <0.05.

### Gene ontology (GO) analysis

Gene lists were uploaded to pantherdb.org^100^ and gene set enrichment was performed using all transcripts expressed during hESC-chondrogensis as a background reference set and ‘GO biological process complete’ as the annotation set. Enrichment was tested using Fisher’s Exact test and significance was corrected using a calculated false discovery rate.

### Co-expression analysis

BioLayout *Express*^3D^ (version 3.3) was used to find highly co-expressed genes and microRNAs (R^2^ > 0.98). Gene transcripts were filtered for protein-coding genes only. Pearson’s correlation coefficients were calculated using a table of normalised read counts for all samples containing both transcript and microRNA reads.

### Protein-protein interaction analysis

Gene list was uploaded to the STRING-db website (Version 10) and high confidence interactions were found (interaction score > 0.7).

### MicroRNA target and transcript factor (TF) target enrichment analysis

Using a custom R script, an exact Fishers Test was performed for each TF and microRNA expressed during the differentiation protocol to identify if it had an enrichment of targets within one or more of the clusters of co-expressed genes. Genes expressed during differentiation were used as a background list. A database of microRNA-target interactions was generated by combining data from TargetScan (7.1) interactions with a total context score < -0.3 (2,332,209 interactions, 995 microRNAs and 15,726 targets) and luciferase validated interactions from miRTarBase (7.0) (3,950 interactions, 451 microRNAs and 1,685 targets). A database of TF-target interactions was generated combining data from TTRUST human transcriptional regulatory interactions (77 TFs; 8215 interactions)^47^, FANTOM5 co-expression data from articular chondrocytes (625 TFs, 174982 interactions) and ENCODE ChIP-Seq data (122 TFs, 44842 interactions)^46^.

### Consent for publication

All authors consent to this publication.

## Supplementary information

Supplementary information

Supplementary information2

Supplementary information3

Supplementary information4

Supplementary information5

Supplementary information6

Supplementary information7

Supplementary information8

Supplementary information9

Supplementary information10

Supplementary information11

Supplementary information12

## Data Availability

All data generated or analysed during this study are included in this published article and its supplementary information files.
