# Supplementary material for: The Transcription Factor-microRNA Regulatory Network during hESC-chondrogenesis
Source: Sci Rep. 2020 Mar 16;10:4744. doi: 10.1038/s41598-020-61734-4 (PMC7075910; doi:10.1038/s41598-020-61734-4)
Supplement: Supplementary file 1 — Supplementary information [file 41598_2020_61734_MOESM1_ESM.pdf]

The Transcription Factor-microRNA Regulatory Network during hESC-chondrogenesis

Rosie Griffiths, Steven Woods, Aixin Cheng, Ping Wang,
Sam Griffiths-Jones, Matthew Ronshaugen, Susan J Kimber.

Supplementary Data Contents

Supplementary Tables:
Table S1 Read Mapping information
Table S2 Normalised Data Tables for transcripts and miRNA
Table S3 Differential Expression Analysis of miRNA
Table S4 Differential Expression Analysis of mRNAs
Table S5 GO-Analaysis-Stage0v3
Table S6 List of genes and miRNAs found within each Co-expression cluster identified using BioLayout
Table S7 Gene Ontology analysis of genes found within each co-expression cluster
Table S8 Fishers Exact Test Results for Transcription Factor target enrichment results within each cluster
Table S9 Fishers Exact Test Results for microRNA target enrichment results within each cluster
Table S10 Differentially Expressed miRNA analysis and Target Enrichment
Table S11 Control vs miR29a inhibitor

Supplementary Figures:
Figure S1 qPCR validation of hESC-chondrogenesis
Figure S2 Spearman's correlation matrix of hESC directed chondrogenesis
Figure S3 PCA of all RNA-seq samples
Figure S4 Differential expression analysis between the intermediate stage and hESCs
Figure S5 qPCR vs. RNAseq comparison
Figure S6 Expression profile of co-expression clusters

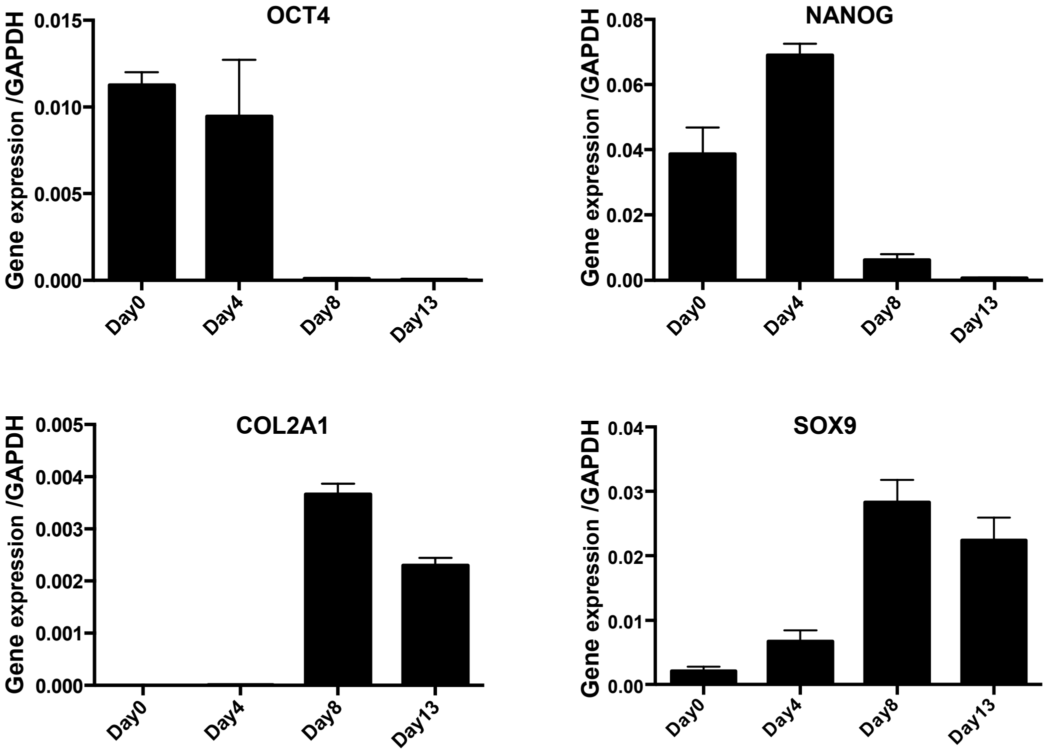

Figure S1 Validation of transcript expression for 2 pluripotency associated and 2 chondrogenic genes for RNAseq runs. Q-PCR analysis of transcript extracted from Man13 cells at 4 stages of chondrogenesis (Oldershaw et al 2010) to validate progression of the protocol. N=3

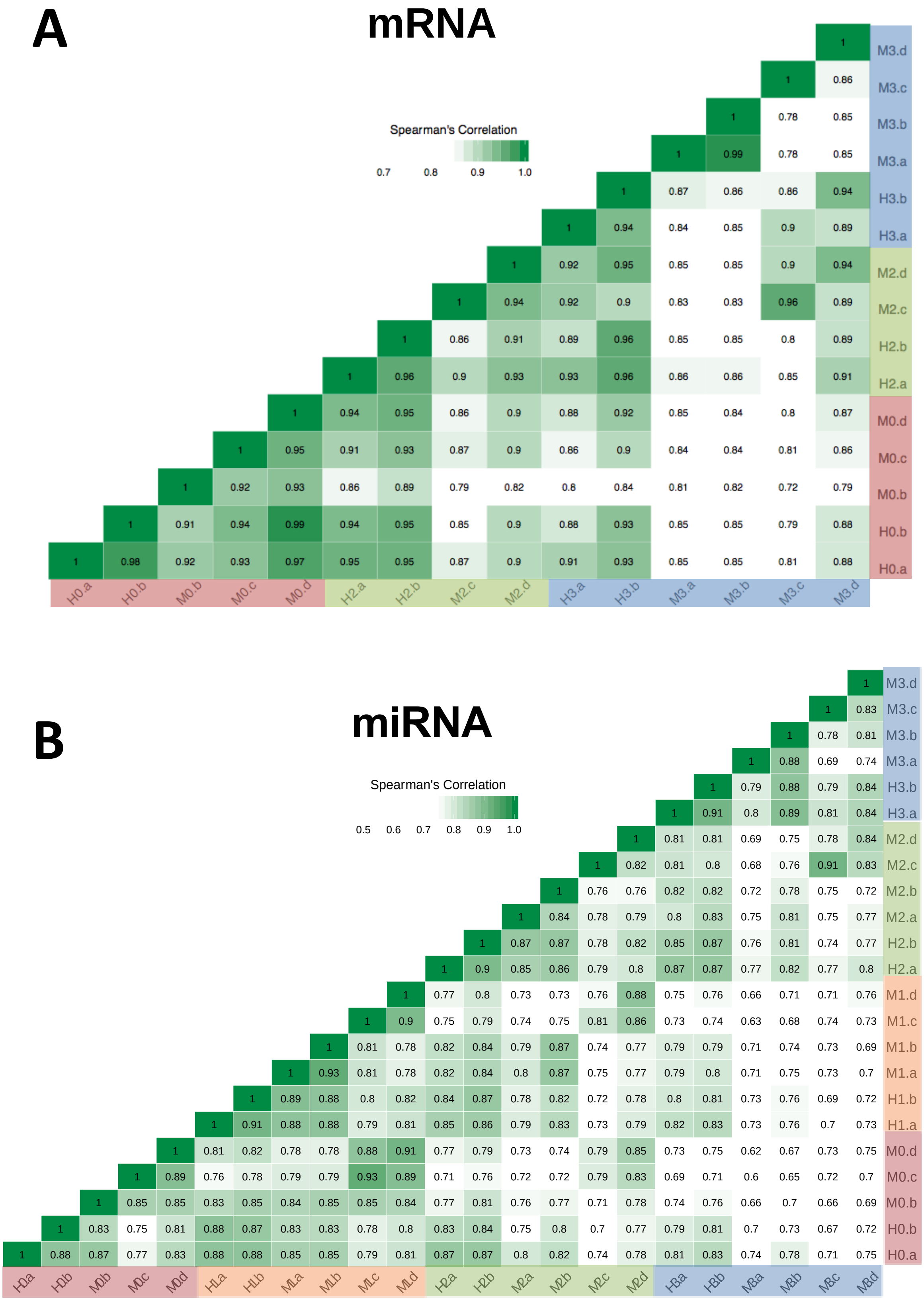

Figure S2 Spearmans correlation matrix of hESC directed chondrogenesis. Spearman's correlation matrix comparing the read counts per million (cpm) of each gene for all mRNA libraries (A) and miRNA libraries (B). Each value refers to the Spearman's correlation coefficient (rho) between the two corresponding samples.

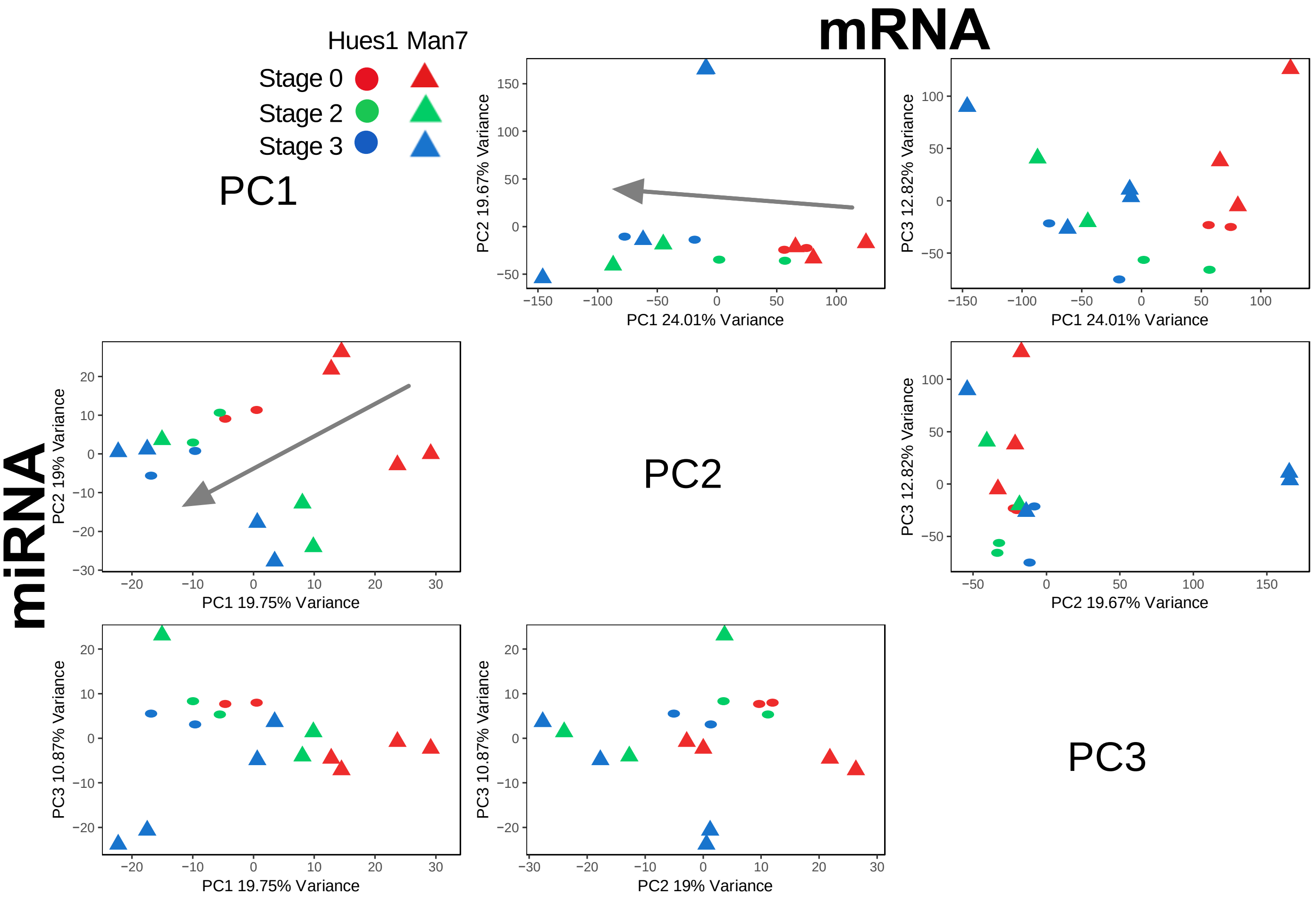

Figure S3 X-Y scatter graphs of PCA scores for top three components (PC1 vs PC2, PC1 vs PC3 and PC2 vs PC3) for microRNA (bottom, left) and mRNA (top, right) log transformed read counts. Samples separate out by stage of the protocol as indicated by the arrows. Points are colour coded by stage of the DDP. PC, principal component.

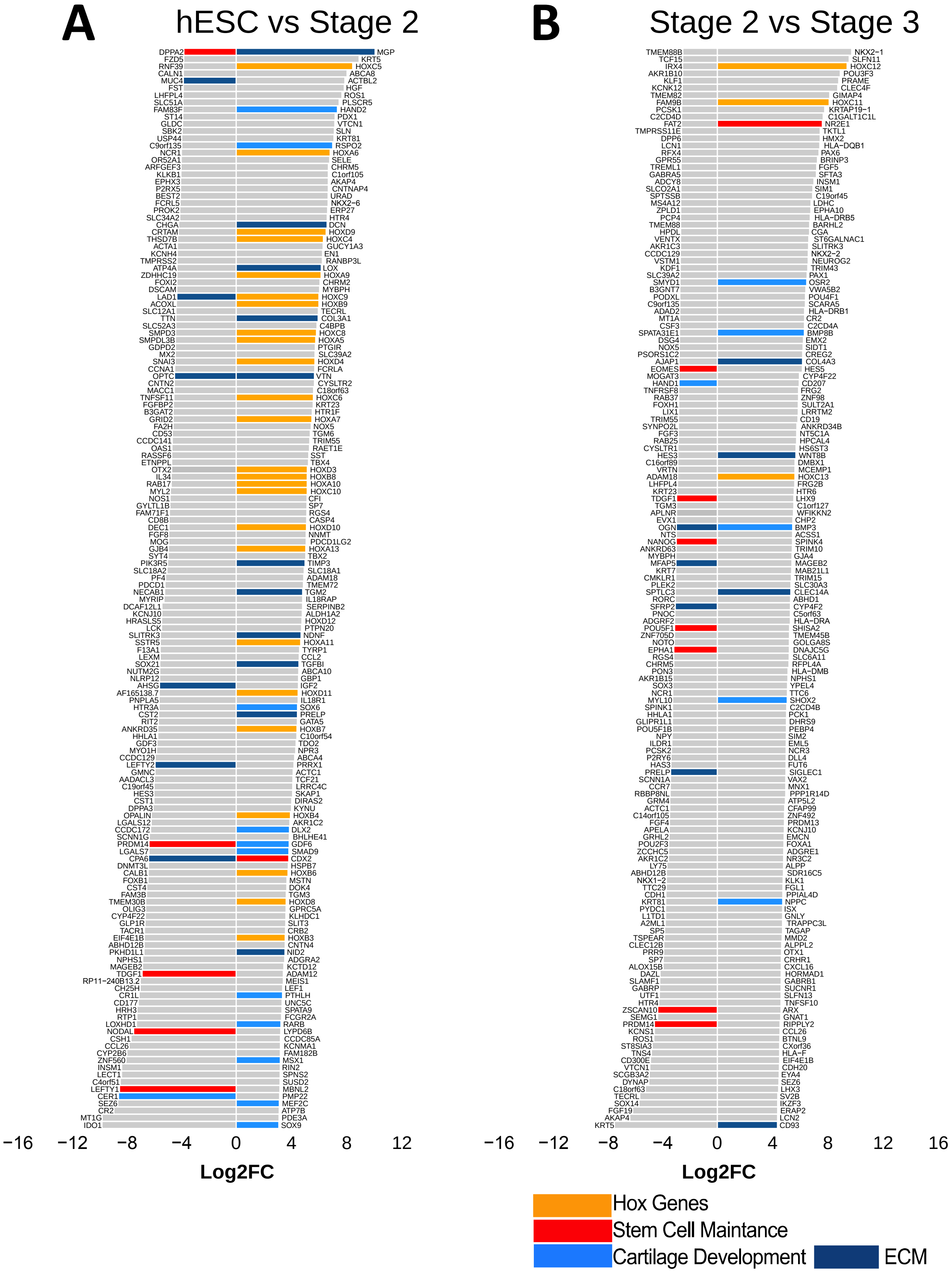

Figure S4 Differential expression analysis between stage 2 and hESCs (A) or Stage 3 (B). Top 150 up-regulated and 150 down-regulated transcripts (Pval<0.05). Some bars are highlighted by function identified from literature.

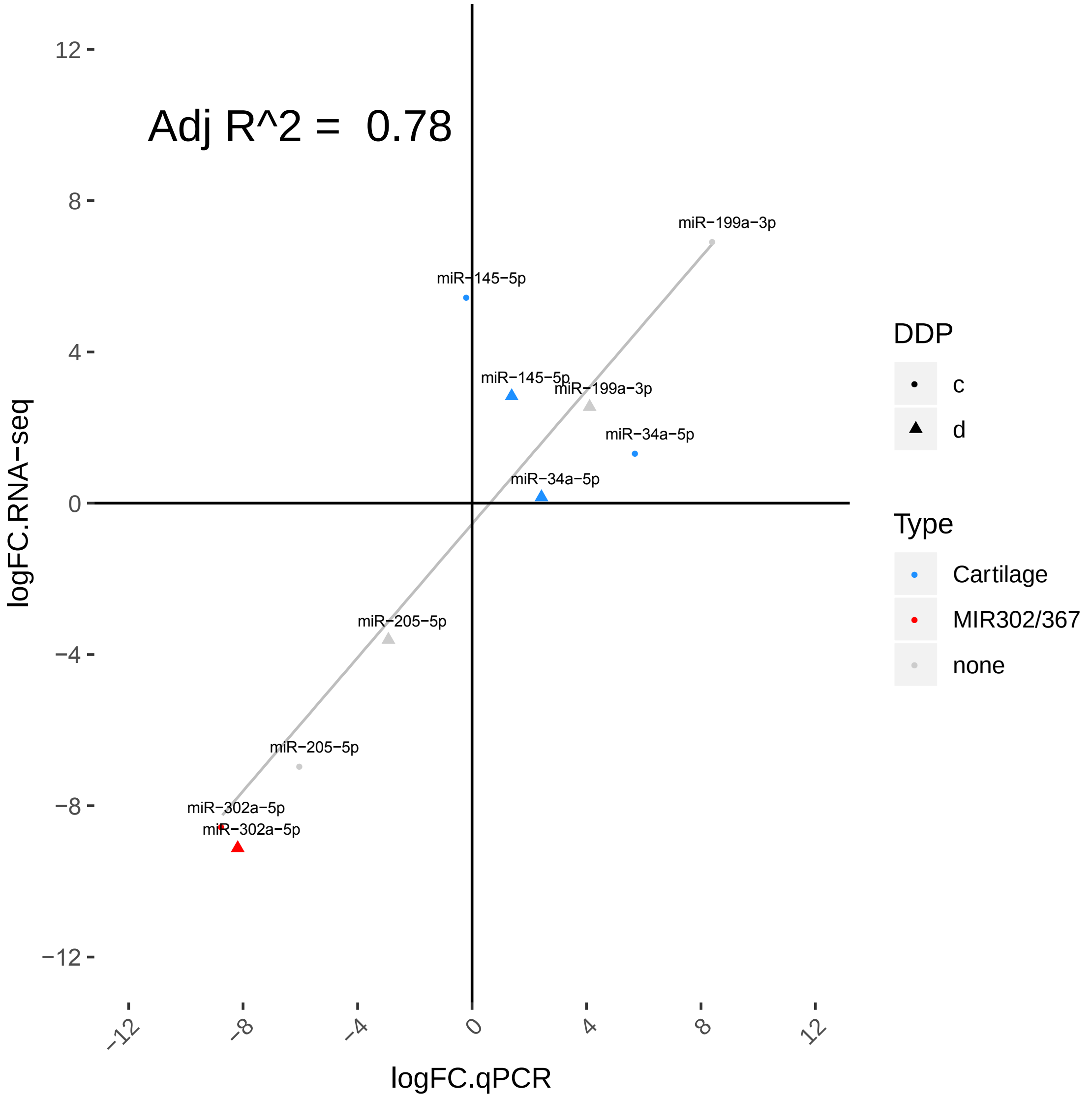

Figure S5 Comparison of qPCR and RNAseq. log2FC values of miRNAs between stage 0 and stage 3 determined by qPCR and RNAseq.

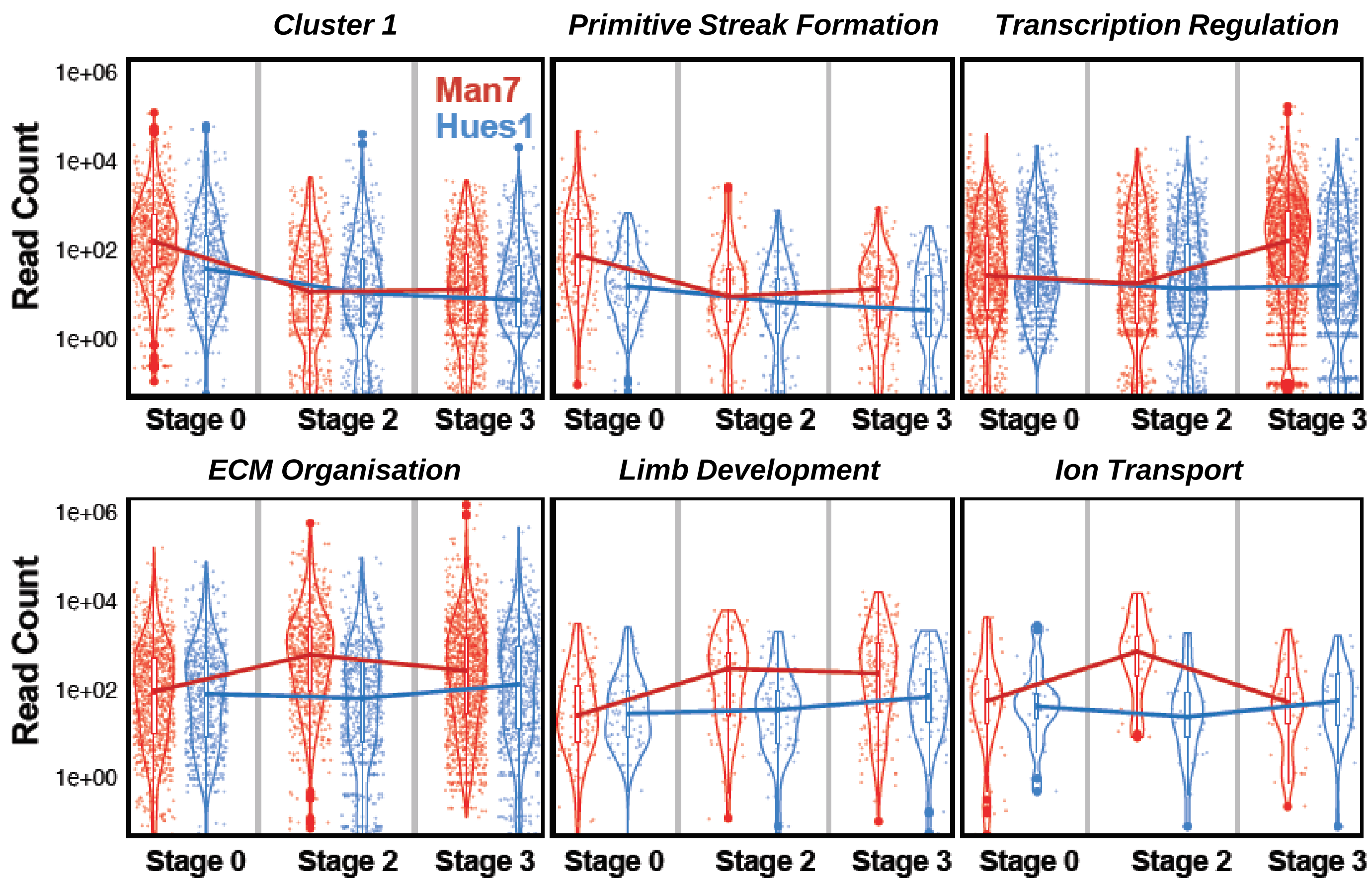

Figure S6 Expression profiles of co-expression clusters generated by BioLayout (Figure 5A). Violin plots of the average read count of each gene/microRNA for all biological replicates (y-axis) in each stage (x-axis). Each cluster is named according to Gene Ontology analysis of genes within the cluster.
